# Modulation of *Klebsiella pneumoniae* Outer Membrane Vesicle Protein Cargo under Antibiotic Treatment

**DOI:** 10.3390/biomedicines11061515

**Published:** 2023-05-24

**Authors:** Aline Castro Rodrigues Lucena, Mariana Galvão Ferrarini, Willian Klassen de Oliveira, Bruna Hilzendeger Marcon, Luis Gustavo Morello, Lysangela Ronalte Alves, Helisson Faoro

**Affiliations:** 1 Laboratory for Applied Science and Technology in Health, Carlos Chagas Institute, FIOCRUZ, Curitiba 81350-010, PR, Brazil; 2 Laboratoire de Biométrie et Biologie Évolutive, UMR 5558, CNRS, Université de Lyon, Université Lyon 1, 69622 Villeurbanne, France; 3 Laboratory for Basic Biology of Stem Cells, Carlos Chagas Institute, FIOCRUZ, Curitiba 81350-010, PR, Brazil; 4 Gene Expression Regulation Laboratory, Carlos Chagas Institute, FIOCRUZ, Curitiba 81350-010, PR, Brazil; 5 CHU de Quebec Research Center, Department of Microbiology, Infectious Disease and Immunology, University Laval, Quebec, QC G1V 0A6, Canada

**Keywords:** *Klebsiella pneumoniae*, antibiotic resistance, outer membrane vesicle, proteomics

## Abstract

*Klebsiella pneumoniae* is a nosocomial pathogen and an important propagator of multidrug-resistant (MDR) and extensively drug-resistant (XDR) strains. Like other Gram-negative bacteria, they secrete outer membrane vesicles (OMVs) that distribute virulence and resistance factors. Here, we subjected a *K*. *pneumoniae*-XDR to subinhibitory concentrations of meropenem, amikacin, polymyxin B, and a combination of these agents to evaluate changes in the protein cargo of OMVs through liquid chromatography–tandem mass spectrometry (LC-MS/MS). Genome sequencing of the clinical isolate *K*. *pneumoniae* strain HCD1 (KpHCD1) revealed the presence of 41 resistance genes and 159 virulence factors. We identified 64 proteins in KpHCD1-OMVs modulated with different antibiotic treatments involved in processing genetic information, environmental information, cell envelope formation, energy metabolism, and drug resistance. The OMV proteome expression profile suggests that OMVs may be associated with pathogenicity, survival, stress response, and resistance dissemination.

## 1. Introduction

Antimicrobial resistance (AMR) is one of the greatest health challenges worldwide, responsible for thousands of deaths per year [1]. *Klebsiella pneumoniae* is a Gram-negative bacterium belonging to the family *Enterobacteriaceae*, usually found in human and animal microbiomes. It is an opportunistic pathogen, which, as well as other ESKAPE organisms, is responsible for most nosocomial infections, with high morbidity and mortality, and with a great potential to “escape” the action of antibiotics [2,3]. In addition, *K*. *pneumoniae* is considered a major source of antibiotic resistance due to the high prevalence of AMR genes (ARGs), contributing to the emergence and spread of multidrug-resistant (MDR) and extensively drug-resistant (XDR) strains [4,5].

Horizontal gene transfer is mainly responsible for the dissemination of ARGs. However, the vesicles produced by bacteria can also transfer resistance factors, including ARGs, small RNAs, and mature proteins [6,7]. Several ARGs have been identified in the vesicles produced by enterobacteria, such as *bla*_*OXA*-*24*_, *bla*_*NDM*-*1*_, *bla*_*CTX*-*M*-*15*_, *aac*(*6*′)-*Ib*-*cr*, and transmitted intra- and interspecies [8,9,10,11,12,13]. Likewise, the *bla*_*KPC*-*2*_ gene has been identified in *K*. *pneumoniae* vesicles and is disseminated by this route, making sensitive strains resistant to imipenem and meropenem [14]. *K*. *pneumoniae*’s secreted vesicles could also disseminate plasmids containing ARGs to different species without phylogenetic correlation, suggesting a role in the development of resistant communities [15].

Gram-negative bacteria produce vesicles from their outer membrane (OM); therefore, they are known as outer membrane vesicles (OMVs) [16,17]. These bilayered structures have a size ranging from 20 to 250 nm and enclose components of the periplasm and OM, such as proteins, toxins, virulence factors, nucleic acids, lipids, and others [16,17,18]. In OMVs isolated from hypervirulent *K*. *pneumoniae*, the ability to transfer virulence factors to a classical *K*. *pneumoniae* strain was observed due to enhanced mucoviscosity and capsule production [19]. In addition, evidence of the role of OMVs produced by hypervirulent *K*. *pneumoniae* in the uptake of iron in an environment with iron was observed, since there was an increase in the production of OMVs in this limiting condition, as well as an increase in the expression of proteins associated with iron uptake [20]. OMVs are mediators of cell survival and pathogenesis and are associated with the secretion and delivery of molecules that regulate the host’s immune response and communication with other bacteria [18,21,22]. The potential to stimulate the inflammatory response of OMVs produced by *K*. *pneumoniae* has already been demonstrated in vitro, with a significant increase in the expression of the pro-inflammatory mediator genes IL-8, IL-6, IL-1β, and TNF-α in human bronchial epithelial cells BEAS- 2, as well as alteration in the miRNA expression profile [23,24]. In vivo, in immunologically normal wild-type C57 mice, the inoculation of OMVs induced by hypervirulent *K*. *pneumoniae* also resulted in increased levels of the proinflammatory chemokines IL-6, IL-8, and TNF-α [25].

The cargo packaging in OMVs is believed to be a selective mechanism and not the result of a random process. This hypothesis is supported by the presence of nucleic acids, differences in the protein content of OM and OMVs, and variations in the number of OMVs produced in response to the environment [21,26,27]. In fact, genes associated with the OMV production process have been identified, demonstrating that this process is genetically regulated [28,29]. In the present study, we evaluate differences in OMVs’ cargo produced by a strain of XDR *K*. *pneumoniae* subjected to treatment with antibiotics of different classes, individually and in combination. We show that treatment with different antibiotics, despite triggering specific changes in OMVs’ cargo, also showed some degree of correlation with protein content. Finally, we present an adapted methodology for obtaining and purifying OMVs from solid culture medium in Petri dishes.

## 2. Materials and Methods

### 2.1. Bacterial Strain and Growth Curves

The *Klebsiella pneumoniae* strain HCD1 (KpHCD1) was isolated from the urine culture of a patient at the Hospital Copa D’Or in Rio de Janeiro, Brazil. The antibiogram indicated resistance to amikacin, amoxicillin, ampicillin, cephalothin, cefepime, ceftriaxone, cefuroxime, Ciprofloxacin, ertapenem, meropenem, nitrofurantoin, norfloxacin, piperacillin, polymyxin B, and sulfamethoxazole, classifying the HCD1 strain as XDR [5]. Minimum inhibitory concentrations (MICs) of meropenem, amikacin and polymyxin B (all from Sigma-Aldrich Brazil, Sao Paulo, SP, Brazil) were obtained according to the guidelines of the Clinical and Laboratory Standards Institute (CLSI), based on broth microdilution in cation-adjusted Mueller–Hinton II broth (BD DIFCO, Lakes, NJ, USA) in 96-well polypropylene microtiter plates. From the MICs, growth curves in the presence of meropenem, amikacin, polymyxin B, and a combination of these antibiotics were performed in triplicate; growth kinetics were recorded every hour for 24 h at 37 °C in a microplate reader (BioTek Synergy H1 Multimode Reader, Agilent, CA, USA).

### 2.2. Genome Sequencing, Assembly, and Annotation

KpHCD1 genomic DNA was isolated and purified with the Wizard Genomic DNA Purification kit (Promega Corporation, WI, USA) according to the manufacturer’s specifications. The whole-genome sequencing was carried out using the Illumina DNA Prep Library Preparation Kit (Illumina, CA, USA) and paired-end sequencing (2 × 300 bp) in the Illumina MiSeq platform. Genome assembly was performed using SPADES v. 3.15.5 [30] and finalized using FGAP [31]. Sequences from potential plasmids were identified using plasmidSPAdes [32]. Protein, rRNA, and tRNA coding regions were identified using Prokka v.1.14.5 [33]. Protein sequences were compared with the CARD database using the RGI tool to identify potential antibiotic resistance genes [34], considering only proteins that obtained “Strict” or “Perfect” hits as being potentially involved in resistance. For virulence factors, the protein sequences were compared against the VFDB database [35] using the BlastP algorithm. The results with an identity higher than 50% and coverage greater than 80% were considered. In the case of multiple results for the same protein, only the alignment with the lowest e-value was considered.

### 2.3. OMV Isolation

The OMVs were isolated from solid medium cultures [36] with adaptations for bacteria growth. KpHCD1 was cultured in Luria–Bertani (LB) broth (BD DIFCO, United States) at 37 °C with shaking. The overnight cultures were diluted and grew to OD600 0.6–0.8. Aliquots of these cell suspensions were then spread onto LB agar plates (90 by 15 mm Petri dishes containing 25 mL of medium) in the absence or at subinhibitory concentrations of antibiotics, determined according to the growth curves performed. The experimental conditions were KP (absence of antibiotics in LB agar plate), MERO (32 µg/mL meropenem added to agar plate), AMI (4 µg/mL amikacin added to agar plate), POL (8 µg/mL polymyxin B added to agar plate), and MAP (12.8 µg/mL meropenem, 1.6 µg/mL amikacin and 3.2 µg/mL polymyxin B added to agar plate), and they were incubated for 16 h at 37 °C to reach confluence. The cells were gently recovered with an inoculation loop and transferred to PBS, which had been previously sterilized by filtering through 0.22 μm pore membranes. Suspended cells were collected via centrifugation at 5000× *g* for 15 min at 4 °C. The supernatants were collected and centrifuged at 15,000× *g* for 15 min at 4 °C to remove debris. The resulting supernatants were filtered through 0.45 μm pore syringe filters and centrifuged at 100,000× *g* for 1 h at 4 °C. Supernatants were discarded, and pellets were resuspended in sterile PBS.

### 2.4. Nanoparticle Tracking Analysis

Nanoparticle tracking analysis (NTA) (Nanosight LM10, Malvern Panalytical, Malvern, UK) was used to determine the OMV concentration and size. The samples were diluted in PBS prior to injection. Videos of 60 s were captured in triplicate. The data were acquired and analyzed using NTA 3.0 (Malvern Panalytical, UK). The total number of OMVs was calculated based on the dilution factors of each condition. Particle size and concentration were analyzed using one-way analysis of variance (ANOVA) followed by Dunnett’s post hoc test using GraphPad Prism software version 9.4.1 (GraphPad Software, San Diego, CA, USA). Differences were considered significant when * *p* < 0.05, *** *p* < 0.001, and **** *p* < 0.0001.

### 2.5. Transmission Electron Microscopy

OMV morphology was verified using transmission electron microscopy (TEM). Purified OMVs were resuspended in PBS and adsorbed onto formvar-coated copper grids. Adsorption was performed by floating the grids on a drop of the OMV suspension for 1 h. The grids were washed in PBS and fixed for 10 min with a solution containing 2.5% glutaraldehyde, 4% paraformaldehyde, and 0.1 M cacodylate buffer. Grids were washed in 0.1 M sodium cacodylate buffer, and negative staining was performed by floating the grids on a drop of 5% uranyl acetate for 1 min. Then, the grids were washed in 18.2Ω water and dried. The samples were analyzed in a Jeol 1400 Plus transmission electron microscope (JEOL, Peabody, MA, USA).

### 2.6. Protein Extraction and Proteomic Analysis

Aliquots from independent replicates of purified OMVs were dried in Speed Vac equipment up to a volume of approximately 15 µL. The same volume of lysis buffer (200 mM DTT, 200 mM Tris. HCl pH 7.5, SDS 6%) was added, and the samples were incubated for 5 min at 95 °C. The protein extracts were centrifuged for 5 min at ~17,500× *g*, and the supernatants were transferred into new tubes. Protein extracts were separated in a 1D-PAGE 13% (*v*/*v*) acrylamide gel. The proteins were reduced with 10 mM DTT, alkylated with 50 mM iodoacetamide, and digested with trypsin. The peptides were desalted with C18 stage tips. Peptides were separated using an online Ultimate 3000 RSLCnano chromatograph (Thermo Fischer Scientific, Waltham, MA, USA) in an analytical column of 15 cm an 75 µm in internal diameter containing particles of C18 of 3 µm in diameter, heated to 60 °C, then analyzed in an Orbitrap Fusion Lumos (Thermo Fischer Scientific, United States) at the mass spectrometry facility RPT02H of the Carlos Chagas Institute—Fiocruz Paraná. The spectra were compared with the database using MaxQuant version 2.0.3.0 [37]. Data were analyzed with the DEP package v1.16.0 [38] in R. Missing values were imputed by the MinProb function (q = 0.01), and differentially expressed proteins were determined considering a threshold for adjusted *p*-values (*p*-adj) of less than 0.05. The functional enrichment of expressed genes for each condition (having a non-zero LFQ value in at least two replicates) and differentially expressed genes (with a less stringent *p*-adj < 0.1) was achieved with the clusterProfiler v4.2.2 package [39].

## 3. Results

### 3.1. KpHCD1 Genome Assembly and Annotation

Genomic DNA was sequenced on the Illumina MiSeq platform. In total, 13,512,820 reads were obtained with 295 bp on average, which encompassed 751 Mpb. With SPADES and FGAP, it was possible to obtain a high-quality draft of the genome of the KpHCD1 strain containing nine contigs, which were ordered using the closest available closed genome as reference (*K*. *pneumoniae* BR21; GenBank GCA_002951595.1). Finally, a single scaffold representing the genome of the KpHCD1 strain was obtained, with a size of 5,278,496 bp and a mean GC content of 57.5%. Eleven contigs potentially derived from plasmids were identified using the plasmiSPAdes. These were separated and submitted to the PLSDB database. Using the plasmid with the highest identity as a reference (Genbank: CP018887), we assembled a plasmid with 100 kbp. However, we could not assemble the other plasmids with the remaining sequences. In the chromosome, 4895 ORFs, 80 tRNAs, five rRNA operons, and 135 potential ncRNAs were identified. Considering the gene content of the plasmids, it was possible to infer that it is a resistance and a conjugative plasmid that carry 135 and 76 ORFs, respectively.

**Figure 1:**
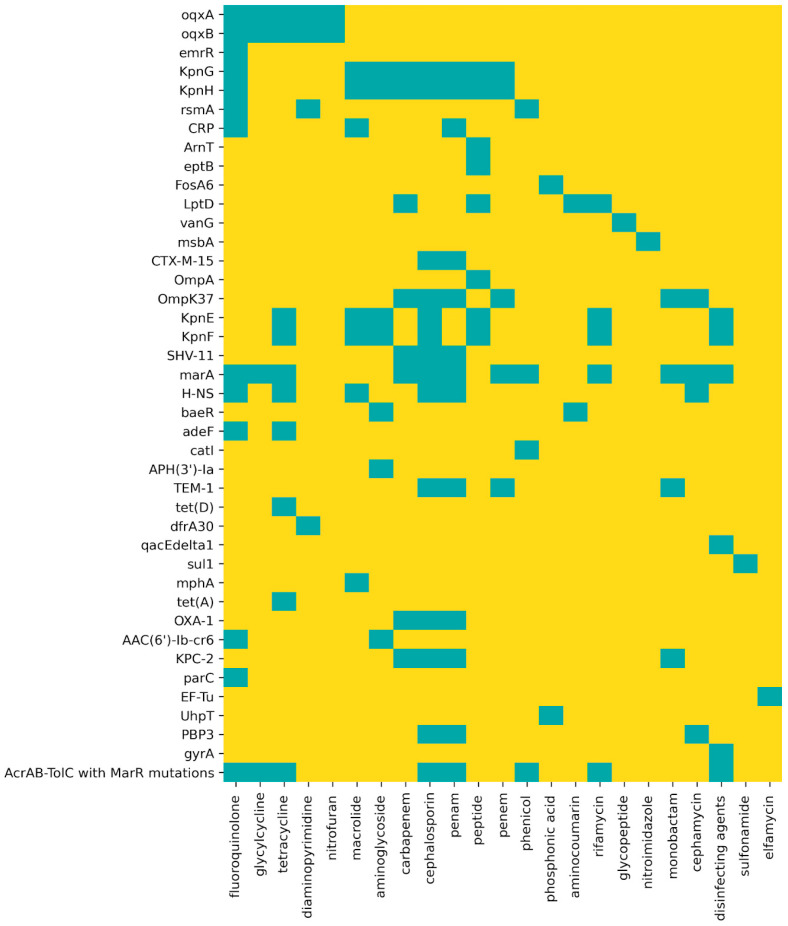
Resistance genes identified in the KpHCD1 genome. Each line contains the genes/proteins associated with resistance mechanisms identified in the genome, and each column contains the different classes of antibiotics. The blue squares indicate the classes of antibiotics to which each gene/protein confers resistance.

Based on the CARD database, 41 genes related to antibiotic resistance were identified (Figure 1) among the annotated ORFs, with distinct mechanisms such as efflux or inactivation of the antibiotic, alteration or substitution of the target, and reduction in permeability to the antibiotic, conferring resistance to several classes of antibiotics. The classes of antibiotics for which the greatest number of resistance genes were found in KpHCD1, considering overlap between classes of antibiotics, were cephalosporin (14 ARGs) and fluoroquinolone (13 ARGs). Based on the VFDB database, we identified 159 virulence factors in the genome of the strain KpHCD1 (Table S1). Among them were capsule, fimbriae, efflux pump, enterobactin, LPS, and biofilm formation.

### 3.2. KpHCD1 Growth Curve in the Presence of Antibiotics

**Figure 2:**
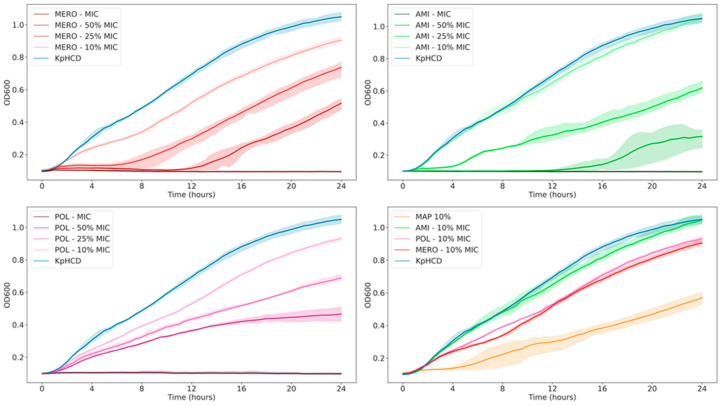
Growth curves of *Klebsiella pneumoniae* KpHCD1 in the presence of different antibiotics.

To evaluate the effect of antibiotics on the KpHCD1 strain, MICs were initially determined using the broth microdilution method. The determined MIC values were 128 mg/mL for meropenem (MERO), 16 mg/mL for amikacin (AMI), and 32 mg/mL for polymyxin B (POL). The measured values were higher than the breakpoints of 8 mg/mL for MERO, 8 mg/mL for AMI, and 2 mg/mL for POL, indicating resistance to these antibiotics [40]. Then, growth curves were obtained, in triplicate, using subinhibitory antibiotic concentrations, which were 25% and 10% of the obtained MIC value (Figure 2). The effects of the presence of the three antibiotics (MAP) at a concentration of 10% of the MIC value were also evaluated. Changes in KpHCD1 growth were observed, with the concentration corresponding to 25% of the MIC value in MERO, AMI, and POL. For MAP, a lower concentration of 10% of the MIC value was used; we observed that the three antibiotics combined have a greater influence on growth than individual antibiotics.

### 3.3. KpHCD1 OMV Characterization

**Figure 3:**
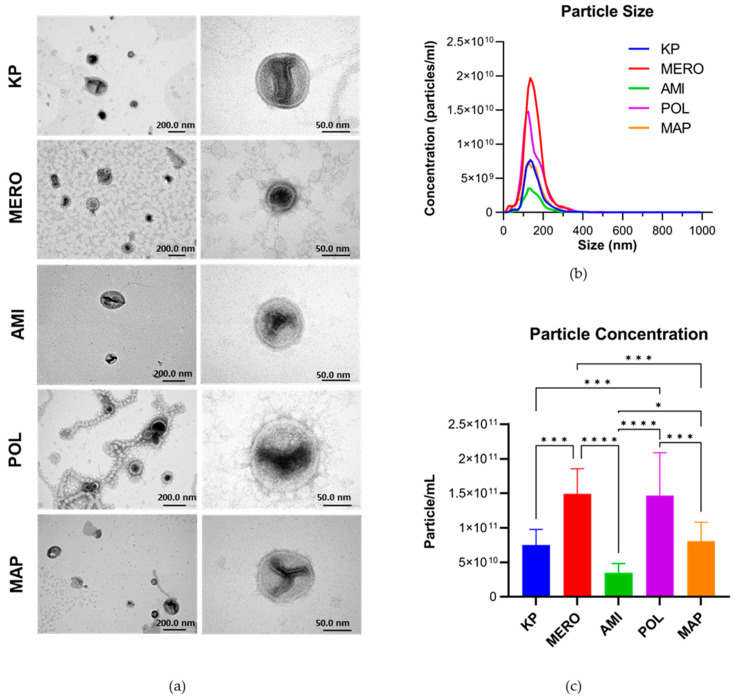
Characterization of *Klebsiella pneumoniae* KpHCD1 OMVs. (**a**) Transmission electron microscopy images of OMVs derived from the KpHCD1 strain with different antibiotic treatments: KP (absence of antibiotics), MERO (32 µg/mL meropenem), AMI (4 µg/mL amikacin), POL (8 µg/mL polymyxin B), and MAP (12.8 µg/mL meropenem, 1.6 µg/mL amikacin, 3.2 µg/mL polymyxin B). (**b**) Graph depicting the correlation between OMV size and the concentration of the particles obtained through nanoparticle tracking analysis of the KpHCD1 OMVs obtained in all conditions; the x-axis represents the particle size (nm) and the y-axis the particle concentration (particles/mL). (**c**) Graph relating the OMV size with the concentration of the particles obtained through nanoparticle tracking analysis of the OMVs obtained in all conditions; the y-axis indicates the concentration of the particles (particles/mL), * *p* < 0.05 *** *p* < 0.001, and **** *p* < 0.0001, using one-way analysis of variance (ANOVA) followed by Dunnett’s post hoc test.

KpHCD1 OMVs were isolated in the absence and presence of antibiotics and then characterized using TEM and NTA. OMVs were observed under all treatment conditions with TEM (Figure 3a). NTA showed that typical spherical OMVs with an average size of 150 nm were obtained in all conditions (Figure 3b). Despite the different concentrations of particles between conditions, the peaks with the highest concentration of particles are around 150 nm. In addition, significant changes were observed in the concentration of isolated particles in each condition (Figure 3c). The particle concentration was similar for MERO and POL; under these conditions, about twice as many OMVs were observed compared to the KP. Interestingly, AMI treatment does not increase the number of OMVs but reduces it to approximately half that of the KP. The combined treatment of the three antibiotics did not result in a significant increase in the production of OMVs in comparison to the KP.

### 3.4. Profiling KpHCD1 OMVs’ Proteome

**Figure 4:**
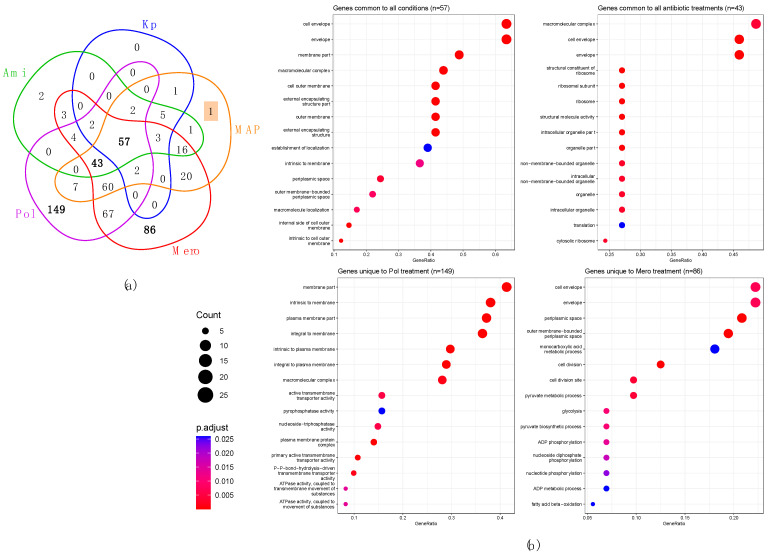
Profile of proteins identified in KpHCD1-OMVs. (**a**) Venn diagram analysis of proteins identified (non-zero LFQ value in at least two replicates within each condition) in KpHCD1-OMVs in treatments with different antibiotics. (**b**) Gene ontology analysis of selected groups: proteins common to all conditions, proteins common to all antibiotic treatments, proteins unique to POL, and proteins unique to MERO.

Proteins were extracted from OMVs isolated from the KpHCD1 strain submitted to treatment with different antibiotics, which were subsequently digested into peptides and analyzed using LC-MS/MS. Considering the proteins identified in at least two replicates of each treatment, 72 proteins were identified in KP, 363 proteins in MERO, 138 proteins in AMI, 393 proteins in POL, and 220 proteins in MAP. Of these proteins, 57 are common to all conditions (Figure 4a). In the presence of antibiotics, 43 proteins are common to AMI, MERO, POL, and MAP. Among the unique proteins, 86 were found in MERO and 149 in POL. Beta-lactamase TEM1 (Kpim_51580) and cold shock protein CspA (Kpim_10710) were identified only in AMI, while the 50S ribosomal protein L18 (Kpim_08500) is unique to MAP. These identified proteins were characterized through gene ontology (GO) analysis (Figure 4b). Among the proteins common to all conditions (*n* = 57, control included), we observed GO-enriched terms for proteins related to the cell envelope, outer membrane (OM), periplasmic space, and inner membrane. Within the set of proteins identified as common to all antibiotic treatments (*n* = 43), we found significant enrichment of GO terms related to the macromolecular complex, cell envelope, molecular structural activity, ribosome, and translation. In POL unique proteins, the enriched terms are related to the membrane, macromolecular complex, active transmembrane transport activity, phosphatase activity, nucleoside-triphosphatase activity, and ATPase activity. Finally, in MERO, unique protein terms related to the cell envelope, periplasmic space, cell division, metabolic process, monocarboxylic acid, pyruvate, glycolysis, and ADP phosphorylation are enriched.

Among the ARGs identified in the KpHCD1 genome (Figure 1), 15 were identified as present in OMVs (Table 1). Considering virulence factors, 27 proteins were identified in KpHCD1-OMVs (Table 2).

Another interesting feature observed in KpHCD1-OMVs is the presence of many ribosomal proteins; in total, 14 30S ribosomal proteins and 21 50S ribosomal proteins were identified (Table S2). The 30S ribosomal proteins S3 and S7 and the 50S ribosomal proteins L1, L6, and L10 were identified in all treatments, including in the absence of antibiotics. The others were identified in the presence of at least one antibiotic. The 30S ribosomal proteins S2, S4, S5, and S10 and the 50S ribosomal proteins L2, L5, L9, L11, L13, and L20 were identified in all treatments with antibiotics. The 50S ribosomal protein L18 was identified only in MAP. The 30S ribosomal protein S18 and the 50S ribosomal proteins L3 and L19 were identified in MERO. Exclusively in POL, the 30S ribosomal proteins S1, S12, S19, S21, and the 50S ribosomal proteins L14 and L29 were identified.

### 3.5. Perturbed Proteins in KpHCD1-OMVs after Treatments with Different Antibiotics

Proteomic analysis of the differentially expressed proteins in KpHCD1-OMVs with different antibiotic treatments showed 64 perturbed proteins (Table S3). All combinations of conditions were analyzed, and an FDR-adjusted p-value (*p*-adj) of less than 0.05 was considered a threshold. Figure S1 depicts the heatmap of these proteins, and Figure S2 shows the GO analysis.

**Figure 5:**
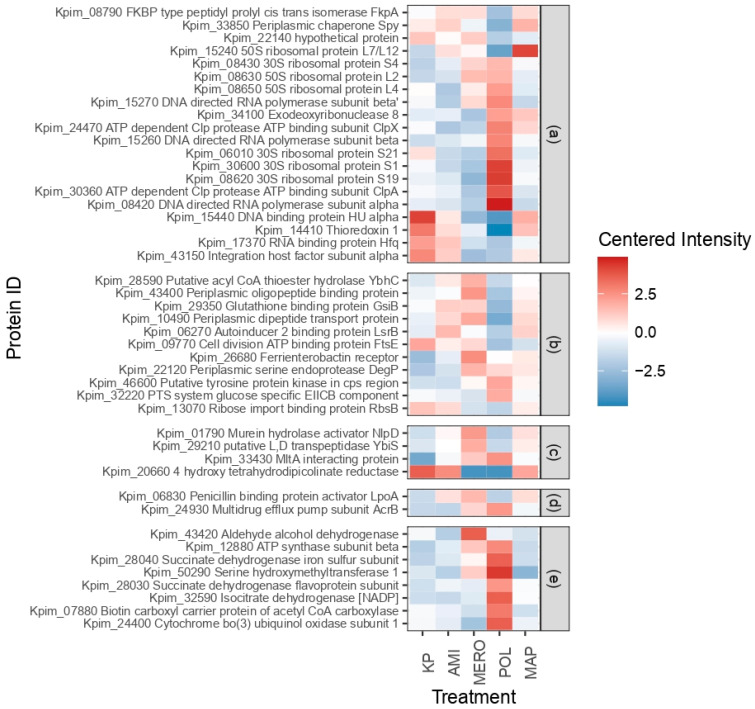
Heatmaps of centered intensity perturbed proteins in KpHCD1-OMVs after treatments with different antibiotics associated with (**a**) processing genetic information, (**b**) environmental information processing, (**c**) cell envelope, (**d**) drug resistance, and (**e**) energy metabolism.

Proteins related to genetic information processing were significantly perturbed in KpHCD1-OMVs after treatments with different antibiotics (Figure 5a). Three DNA-directed RNA polymerase alpha and beta subunits are up-regulated in POL. DEPs’ ribosomal proteins present an up-regulated profile in POL, except for the 50S ribosomal protein L7/L12, which is up-regulated in MAP and down-regulated in POL. The 50S ribosomal protein L2 is up-regulated in MERO and POL. ATP-dependent Clp protease ATP binding subunits ClpX and ClpA are components of the ClpA–ClpP complex, whose primary function is the degradation of unfolded proteins. Both have their expression up-regulated in POL, whereas ClpA is down-regulated in MERO and ClpX is down-regulated in AMI and MERO. FKBP-type peptidyl-prolyl cis-trans isomerase FkpA (FkpA), a possible chaperone of envelope proteins in the periplasm, is down-regulated in POL and up-regulated in other treatments. Thioredoxin 1 participates in several biological processes, including transcription regulation, and is down-regulated in POL. The periplasmic chaperone Spy (Spy) is up-regulated in MAP and AMI. The RNA binding protein Hfq (Hfq) and DNA binding protein HU alpha are down-regulated in POL and MERO. Exodeoxyribonuclease 8 is up-regulated in MAP and POL.

Antibiotic treatments also perturb proteins associated with environmental information processing in KpHCD1-OMVs (Figure 5b). Periplasmic dipeptide transport protein, periplasmic oligopeptide binding protein, putative acyl CoA thioester hydrolase YbhC, and ferrienterobactin receptor are up-regulated in MERO. The periplasmic serine endoprotease DegP (DegP) changes its function in response to changes in temperature, being a chaperone at low temperatures and a peptidase at high temperatures, and also has its expression increased in MERO. The glutathione binding protein GsiB is part of an ABC transporter complex involved in the import of glutathione and is up-regulated in MERO and AMI. Autoinducer 2 binding protein LsrB is also up-regulated in AMI. The PTS system-specific glucose EIICB component and putative tyrosine protein kinase in the cps region are up-regulated in POL, while the cell division ATP binding protein FtsE and ribose import binding protein RbsB are down-regulated in POL.

Some proteins associated with the cell envelope have their expression increased in MERO (Figure 5c). Among them are the murein hydrolase activator NlpD required for septal murein cleavage in cell division, the putative L, D transpeptidase YbiS, which anchors the main OM lipoprotein, and the MltA interacting protein, which forms a complex acting in the enlargement and septation of the murein sacculus. In contrast, 4 hydroxy tetrahydro picolinate reductase is up-regulated in AMI, KP, and MAP and down-regulated in MERO and POL. This enzyme is related to the production of components used to bind peptidoglycan and the cell wall.

Among the perturbed proteins associated with drug resistance (Figure 5d), the penicillin-binding protein activator LpoA, involved in the regulation of beta-lactamases, is up-regulated in MERO, AMI, and MAP. In contrast, the multidrug efflux pump subunit AcrB is up-regulated in POL and MERO. In general, the polymyxin treatment perturbs proteins associated with energy metabolism (Figure 5e). They are up-regulated in POL, except for aldehyde alcohol dehydrogenase, which is up-regulated in MERO. ATP synthase subunit beta and serine hydroxymethyltransferase are also up-regulated in MERO.

## 4. Discussion

Interaction with the host’s immune system, communication with other bacteria, and the dispersion of ARGs are some of the functions attributed to OMVs [17,21]. In the present study, we used quantitative proteomics to analyze the protein content of OMVs produced by the *K*. *pneumoniae* KpHCD1 strain, an XDR clinical isolate treated with different antibiotics with distinct mechanisms of action, as well as with combinations of these. Meropenem leads to the inhibition of bacterial growth or lysis by binding and inactivating Penicillin-binding proteins (PBPs) that are involved in peptidoglycan metabolism; on the other hand, amikacin leads to the mistranslation of proteins by binding to the 30S subunit, altering the conformation of the A site and impairing the proofreading capabilities of the ribosomes. Finally, polymyxin, which reduces the OM integrity by binding to lipid A, destabilizes lipopolysaccharide (LPS) and has been proposed as a last resort for the treatment of MDR pathogens [41,42,43,44]. We observed an increase in the production of OMVs from the KpHCD1 strain in the MERO and POL treatments, both antibiotics that act on the cell wall. This hypervesiculation profile with meropenem and polymyxin B treatments was also detected in *E*. *coli* [45]. In the same study, the authors also found that treatment with fosfomycin, another antibiotic that acts on the cell wall, leads to an increase in the production of OMVs. In *P*. *aeruginosa* and *Elizabethkingia anophelis*, an increased production of OMVs was also demonstrated in meropenem treatments [46,47], whereas a decreased production was found in amikacin treatments. Amikacin interferes in protein synthesis, and in *E*. *coli* O104:H4, other antibiotics whose mechanism of action works on protein synthesis (rifamixin, tigecycline, azithromycin, and chloramphenicol) have been shown to reduce or not change the production of OMVs compared to antibiotic-free conditions [45].

We identified 41 ARGs and 159 virulence factors in the KpHCD1 strain genome. We detected three beta-lactamases, two ESBLs (TEM1 and CTX-M-15), and one carbapenemase (KPC-2), and it is suggested that the acquisition of ESBL-encoding plasmids is related to an increase in the virulence potential of the strain [48]. We also detected two outer membrane proteins, OmpA and OmpK37, which contribute to resistance and virulence. The absence of OmpK36 coupled with the expression of beta-lactamases is related to high levels of resistance to carbapenems, especially the ESBL-positive strains [48,49]. Among the virulence factors, type 1 and type 3 fimbriae genes were identified in the KpHCD1 genome. They are important mediators of *K*. *pneumoniae* adhesion, and type 3 is also associated with biofilm formation [50,51]. Several genes for enterobactin have also been identified in the KpHCD1 genome. Enterobactin is considered the primary iron uptake system in *K*. *pneumoniae* infection, with its expression almost ubiquitous in *K*. *pneumoniae* [51,52]. The efflux pumps AcrAB and OqxAB, which mediate the resistance to various antibiotics and virulence were also detected [48,53].

The products of approximately one third of the ARGs identified in the KpHCD1 strain genome were detected in OMVs produced by the KpHCD1 strain in the presence or absence of antibiotics. Among these proteins, two components of the Lpt machinery have been identified, LptD and MsbA. LptD was identified in all conditions in which antibiotics were used; in the present study, MsbA was identified in MERO, POL, and MAP conditions—the mechanisms of action of these antibiotics act on the cell wall. LptD and LptE form a hetero-oligomeric complex responsible for translocating LPS to the OM and correctly assembling it on the surface of the cell wall [54,55]. Hashemi suggested that the exposure of *P*. *aeruginosa* to chlorhexidine leads to the up-regulation of LptD, resulting in decreased OM permeability [56]. MsbA is responsible for transporting lipid A from LPS to the outer leaflet of the OM [57]. Therefore, the presence of these proteins in OMVs can result from an increase in the production of this protein and accumulation in the OM as a response to the stress caused by antibiotics that act on the cell wall; however, intriguingly, LptD has also been identified in AMI.

ArnT was another LPS modification-related protein found in OMVs in the presence of antibiotics. ArnT is a transferase that adds an L-Ara-N molecule to the lipid A portion of LPS. This modification confers resistance to polymyxin [58,59]. In our data, this protein was identified in OMVs produced by the KpHCD1 strain treated with polymyxin B. The presence of this protein in OMV may be associated with the increase in ArnT expression and forwarding to the periplasmic space. Hussein observed that this protein is down-regulated in *K*. *pneumoniae* polymyxin-susceptible OMVs after treatment with polymyxin B when compared to *K*. *pneumoniae* polymyxin-resistant OMVs, as well as other LPS-modifying enzymes, and proposed that the bacteria generating OMVs with unmodified LPS could be a more attractive target for the antibiotic, acting as a decoy and, thus, protecting the cell [60]. However, exporting ArnT via OMVs could provide some protection mechanism for nearby cells, disseminating the mechanism that generates resistance.

The OM proteins OmpA and OmpK37 were identified in OMVs at a high intensity under all treatment conditions. In *K*. *pneumoniae*, OmpA is related to virulence through protection against the innate immune response [50]. The deletion of OmpA results in increased susceptibility to several antibiotics in *A*. *baumanii* and *E*. *coli* [61,62]. However, OmpA is required for the systemic dissemination of pulmonary infection by *A*. *baumanii* in mice and is associated with the host immune response [63,64]. In addition to this function, Kim proposed that the beta-lactamase in the lumen of the OMV can hydrolyze beta-lactams and that these antibiotics enter the OMV via the porins [65]. In our results, the KPC-2 carbapenemase appears under all treatment conditions, suggesting a constitutive expression independent of the presence of antibiotics. OMVs containing active carbapenemases can spread to neighboring cells, making them resistant, regardless of whether they carry resistance genes.

We identified many ribosomal proteins in the OMVs of the KpHCD1 strain. The 30S subunit comprises the 16S rRNA and 21 ribosomal proteins [66]; we identified 15 proteins in our data. The 50S subunit has the 5S rRNA, 23S rRNA, and 33 ribosomal proteins [66], of which we identified 21 in OMVs. In addition, the antimicrobial activity of peptides derived from L1 from *Helicobacter pylori* in *E*. *coli* and *Bacillus megaterium* and also from proteins L27 and L37 from *Lactobacillus salivarius* against *Streptococcus pyogenes*, *S*. *uberis*, and *Enterococcus faecium* has already been demonstrated [67,68,69], indicating that their presence in OMVs may be associated with competition with other pathogens.

Transcription and translation processes seem to be affected by the presence of polymyxin B, which is reflected in the proteins expressed in OMVs. Polymyxin B and its analogs have been shown to bind to 16S rRNA but do not interfere with translation [70]. In our data, Hfq was not identified in POL. The Hfq protein facilitates the correct processing and folding of mature 16S rRNA, and its inactivation leads to a decrease in the number of mature ribosomes in *E*. *coli* [71]. Perhaps polymyxin B inactivates Hfq, influencing ribosome biogenesis and justifying the amount of up-regulated ribosomal proteins identified in OMV. In addition, the absence of Hfq in *K*. *pneumoniae* deregulates the expression of numerous genes [72]. In our data, the RNA polymerase sigma factor RpoD was identified only in OMVs treated with POL, and RNA polymerase alpha and beta subunits were up-regulated with the same treatment. The presence of Hfq in the OMVs under the other treatment conditions may be associated with the virulence of the KpHCD1 strain since the absence of Hfq prevents the dissemination of *K*. *pneumoniae* in extra-intestinal organs and attenuates systemic infection in mice [72].

The modulation of periplasmic chaperones DegP, FkpA, and Spy in OMVs is intriguing. These proteins are associated with the biogenesis and homeostasis of OMPs and are essential for bacterial survival under stress [73,74,75]. Interestingly, these proteins are present in the OMVs since they are necessary for the bacterium in stressful situations. In addition, the deletion of DegP in *Meiothermus ruber* increases the production of OMVs, which is likely associated with an increase in the number of denatured proteins [76]. Analyzing the expression of these proteins in the cell during exposure to antibiotics would be interesting and help better understand the role they may be playing in OMVs.

As mentioned before, like other carbapenems, MERO interferes with cell wall synthesis. Inside the bacteria, it binds to PBPs and inactivates them, preventing them from completing the transpeptidation of peptidoglycan strands, inhibiting growth, and resulting in cell death [77]. In *E*. *coli*, the absence of TolA leads to increased vesiculation and enrichment of NlpD and Mlta in OMVs [78]. Reimer suggests that this hyper-vesiculation results from incomplete binding between the inner and OM [78]. Tsang proposed that the Tol-Pal system promotes the invagination of the OM at the site of cell division, and the cell remodeling appears to be coordinated, in part, by coupling NlpD activation with OM invagination [79]. We also observed an increase in vesiculation in MERO and up-regulated cell envelope-associated proteins such as NlpD, YbiS, MltA, LpoA, and TolB, showing that this machinery affected by MERO is directly related to the production of OMVs.

We observed changes in proteins associated with up-regulated energy metabolism in POL. Metabolomic analysis of the killing effect of polymyxin-B-enrofloxacin on *P*. *aeruginosa* shows disturbances in lipid, carbohydrate, nucleotide, and energy metabolism [80]. The modulation of these proteins in OMVs may result from changes in the cell caused by exposure to the antibiotic. Therefore, an analysis of variations in the expression of cell proteins can help to better understand these proteins packaged in OMVs.

We found that the multidrug efflux pump subunit AcrB is up-regulated in MERO and POL treatments. This efflux pump is associated with resistance and virulence in *K*. *pneumoniae* [53]. The presence of efflux pumps in OMVs may be associated with the sequestration of antibiotics from the extracellular milieu [81]. In addition, the deletion of *acrAB* results in increased susceptibility of *E*. *coli* to polymyxin B [82]. The increase in expression we observed in vesicles was greater in POL, perhaps because this pump is overexpressed in the cell as a response to the presence of the antibiotic.

## 5. Conclusions

We sequenced the genome of the *K*. *pneumoniae* HCD1 strain and detected several ARGs that confer resistance to several classes of antibiotics, as well as several of the key virulence factors of *K*. *pneumoniae*. We also describe a methodology for OMV isolation and purification, already used in fungi, from the cultivation of bacteria in solid medium, in Petri dishes containing the culture medium and the different antibiotics used in this work. From the LC-MS/MS analysis of the protein content of OMVs produced by the KpHCD1 strain under different antibiotic treatment conditions, we observed changes in the expression of proteins. Among the disturbed proteins due to the antibiotic treatments, we were able to infer several mechanisms through which OMVs can act, such as pathogenicity, survival, stress response, and resistance dissemination. The bacterium seems to use OMVs in different ways to protect itself from the action of antibiotics, as well as to contribute to the bacterial community, corroborating the principle that there is a selective packing of OMVs and it is not a simple random process.

## Figures and Tables

**Table 1:** Products of ARGs identified in KpHCD1-OMVs in the different treatments.

Protein IDs	Protein Name	ARGs	Intensity ^1^	Treatment ^2^
Kpim_31130	Outer membrane protein A	OmpA	3.25 × 10^11^	All
Kpim_36000	Outer membrane protein N	OmpK37	1.38 × 10^11^	All
Kpim_15180; Kpim_08710	Elongation factor Tu 1	EF-Tu	1.96 × 10^10^	All
Kpim_53330	Carbapenem-hydrolyzing beta-lactamase KPC	KPC-2	5.39 ×10^9^	All
Kpim_20790	LPS-assembly protein LptD	LptD	3.67 × 10^9^	MERO, AMI, POL, MAP
Kpim_00390	Efflux pump periplasmic linker BepF	oqxA	1.69 × 10^9^	MERO, AMI, POL, MAP
Kpim_00400	Efflux pump membrane transporter BepE	oqxB	1.13 × 10^9^	MERO, POL, MAP
Kpim_30630	Lipid A export ATP-binding/permease protein MsbA	msbA	8.35 × 10^8^	MERO, POL, MAP
Kpim_08900	cAMP-activated global transcriptional regulator CRP	CRP	6.86 × 10^8^	MERO
Kpim_43440	DNA-binding protein H-NS	H-NS	3.00 × 10^8^	POL, MAP
Kpim_09910	Undecaprenyl phosphate-alpha-4-amino-4-deoxy-L-arabinose arabinosyl transferase	ArnT	1.75 × 10^8^	POL
Kpim_51580	Beta-lactamase TEM	TEM-1	1.50 × 10^8^	AMI
Kpim_30850	Beta-lactamase CTX-M-1	CTX-M-15	1.17 × 10^8^	POL
Kpim_21190	Peptidoglycan D, D-transpeptidase Ftsl	PBP3	1.13 × 10^8^	MERO
Kpim_05620	DNA topoisomerase 4 subunit A	parC	1.50 × 10^7^	MERO

^1^ Summed intensity of all peptides referring to the protein. ^2^ Treatment having a non-zero LFQ value in at least two replicates.

**Table 2:** Virulence factors identified in KpHCD1-OMVs in the different treatments.

VF * Category	Protein Ids	VF * Name	Protein Name	Intensity ^1^	Treatment ^2^
Adherence	Kpim_08900	Type IV pili	cAMP activated global transcriptional regulator CRP	6.86 × 10^8^	MERO
	Kpim_15180	EF-Tu	Elongation factor Tu-1	1.96 × 10^10^	All
	Kpim_16990	Hsp60	60 kDa chaperonin	4.09 × 10^9^	All
	Kpim_22480	IlpA	D methionine binding lipoprotein MetQ	5.29 × 10^9^	MAP, MERO, POL
Antimicrobial activity/Competitive advantage	Kpim_08190	AcrAB	Multidrug efflux pump subunit AcrA	2.33 × 10^10^	All
	Kpim_08200	AcrAB	Multidrug efflux pump subunit AcrB	1.26 × 10^10^	All
Biofilm	Kpim_00400	AdeFGH efflux pump	Efflux pump membrane transporter BepE	1.13 × 10^9^	MAP, MERO, POL
	Kpim_03480	Type 3 fimbriae	Outer membrane usher protein HtrE	8.66 × 10^7^	MAP, MERO
	Kpim_03500	Type 3 fimbriae	Hypothetical protein	6.30 × 10^8^	AMI, MAP, MERO, POL
Immune modulation	Kpim_11280	LOS	3 deoxy D manno octulosonic acid transferase	1.10 × 10^8^	POL
	Kpim_30630	LOS	Lipid A export ATP binding/permease protein MsbA	8.35 × 10^8^	MAP, MERO, POL
	Kpim_31890	LOS	Lipid A biosynthesis lauroyltransferase	1.21 × 10^8^	POL
	Kpim_32150	LPS	Acyl carrier protein	4.38 × 10^8^	KP, MAP
	Kpim_43450	LOS	UTP glucose 1 phosphate uridylyltransferase	1.59 × 10^8^	MAP, MERO
	Kpim_46450	Capsule	6 phosphogluconate dehydrogenase, decarboxylating	2.12 × 10^7^	MERO
	Kpim_46600	Capsule	Putative tyrosine protein kinase in cps region	2.87 × 10^9^	MAP, MERO, POL
	Kpim_46610	Capsule	Hypothetical protein	6.01 × 10^9^	AMI, MAP, MERO, POL
	Kpim_46620	Capsule	Hypothetical protein	5.28 × 10^9^	All
Invasion	Kpim_31130	OmpA	Outer membrane protein A	3.25 × 10^11^	All
Nutritional/ Metabolic factor	Kpim_09560	GGT	Glutathione hydrolase proenzyme	9.62 × 10^8^	AMI, MERO
	Kpim_18230	MgtBC	Magnesium transporting ATPase, P type 1	4.29 × 10^8^	MAP, MERO, POL
	Kpim_20690	Pyrimidine biosynthesis	Carbamoyl phosphate synthase large chain	7.81 × 10^8^	POL
	Kpim_26680	Ent	Ferrienterobactin receptor	1.42 × 10^10^	AMI, MAP, MERO, POL
	Kpim_32310	Aerobactin	Ferric aerobactin receptor	2.93 × 10^10^	All
Regulation	Kpim_01780	RpoS	RNA polymerase sigma factor RpoD	8.44 × 10^8^	POL
	Kpim_32500	PhoPQ	Sensor protein PhoQ	2.14 × 10^8^	POL
Stress survival	Kpim_51330	ClpC	ATP-dependent Clp protease ATP-binding subunit ClpC	4.07 × 10^8^	MAP, MERO, POL

* Virulence factor. ^1^ Summed intensity of all peptides referring to the protein. ^2^ Treatment having a non-zero LFQ value in at least two replicates.

## Data Availability

*Klebsiella pneumoniae* KPHCD1 genome accession number JARBIY000000000.

## Supplementary Materials

The following supporting information can be downloaded at: https://www.mdpi.com/article/10.3390/biomedicines11061515/s1, Figure S1: Heatmap of centered intensity of all perturbed proteins in KpHCD1-OMVs after treatments with different antibiotics; Figure S2: Gene ontology analysis of all perturbed proteins in KpHCD1-OMVs after treatments with different antibiotics; Table S1: Virulence factors identified in the KpHCD1 genome; Table S2: Ribosomal proteins identified in KpHCD1-OMVs after treatments with different antibiotics; Table S3: Perturbed proteins in KpHCD1-OMVs after treatments with different antibiotics.
