# Supplementary material for: Modulation of Klebsiella pneumoniae Outer Membrane Vesicle Protein Cargo under Antibiotic Treatment
Source: Biomedicines. 2023 May 24;11(6):1515. doi: 10.3390/biomedicines11061515 (PMC10294825; doi:10.3390/biomedicines11061515)

Figure S1. Heatmaps of centered intensity of all perturbed proteins in KpHCD1-OMVs after treatments with different antibiotics.

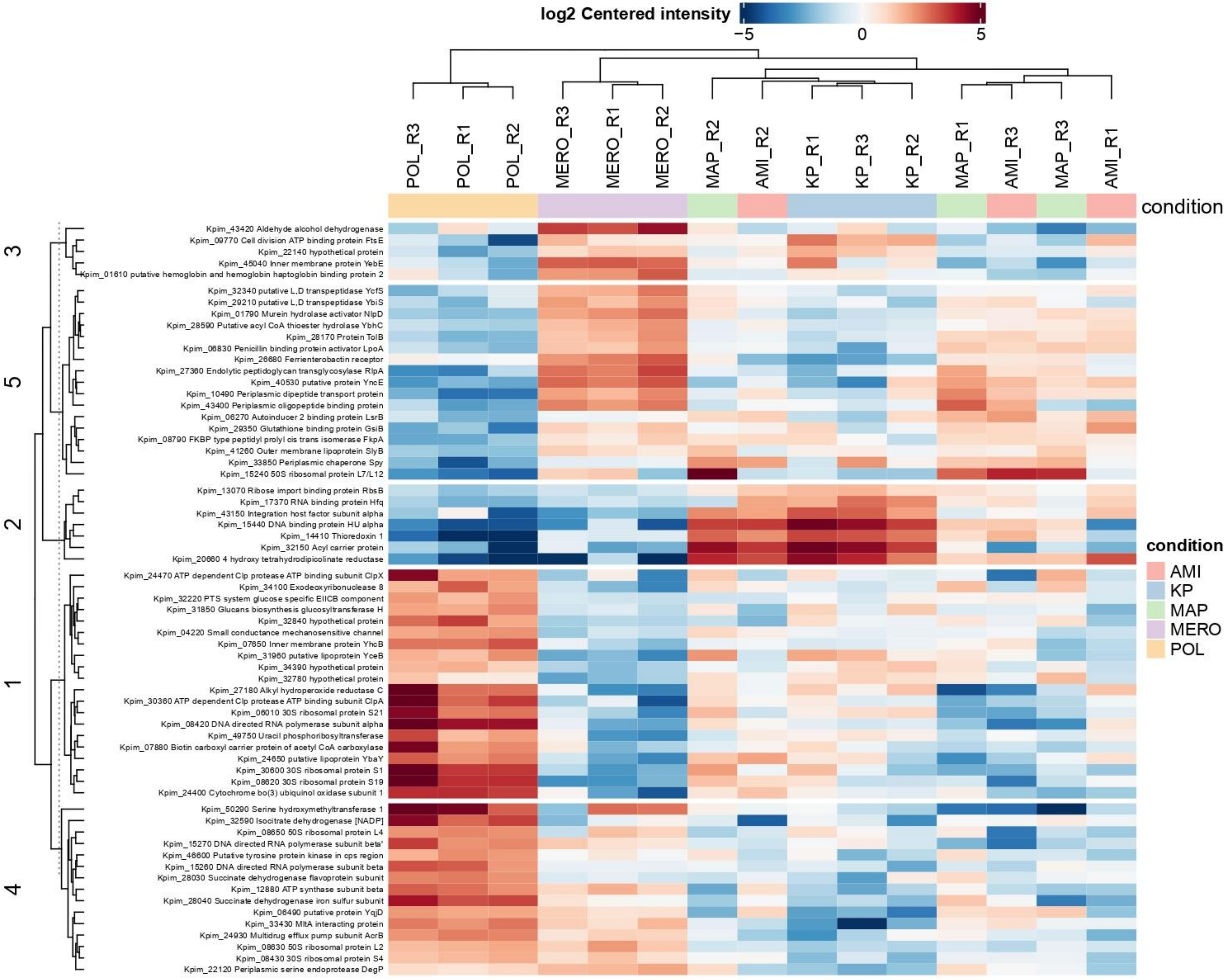

Figure S2. Gene Ontology analysis of all perturbed proteins in KpHCD1-OMVs after treatments with different antibiotics.

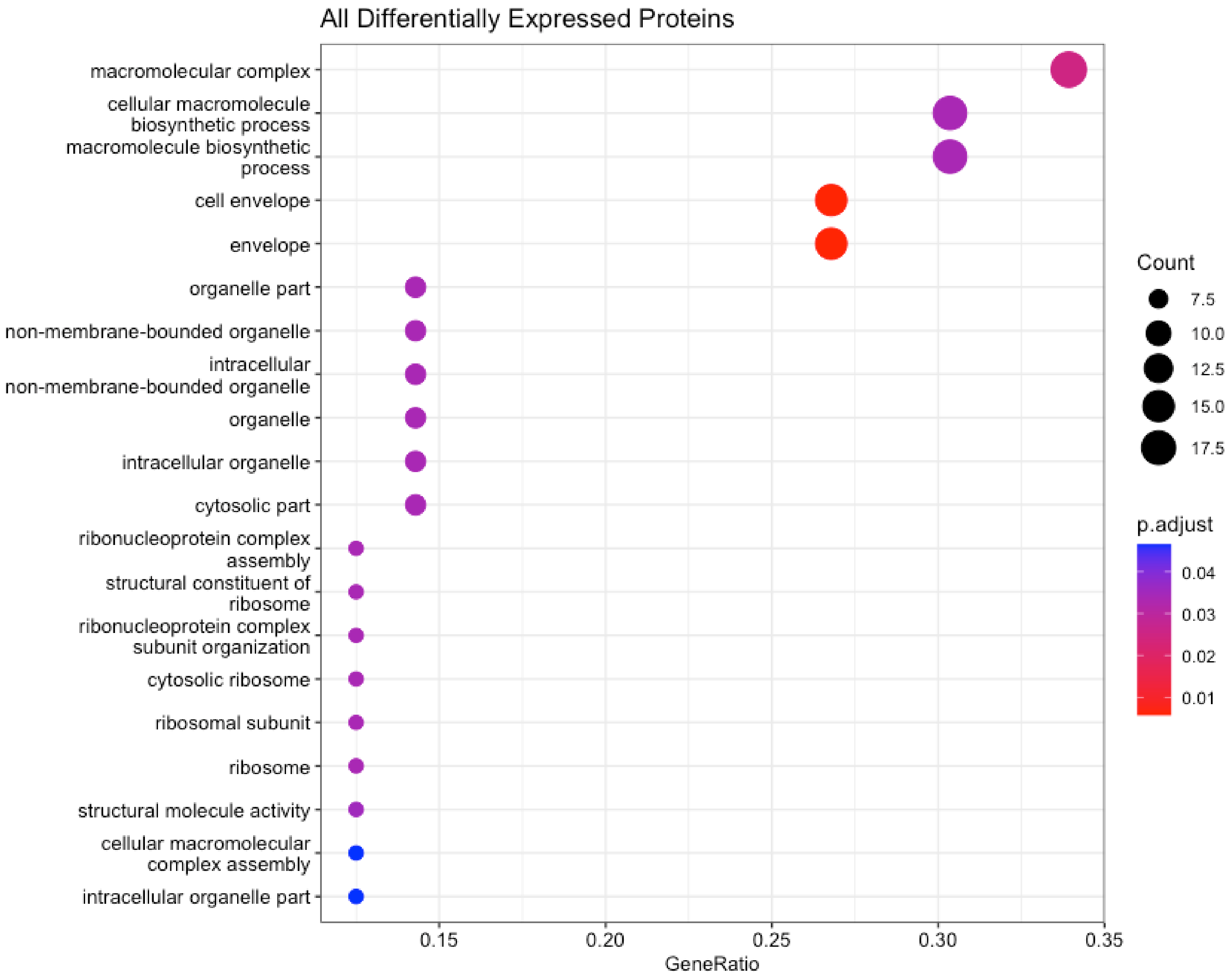

Supplement: Supplementary file 1 [file biomedicines-11-01515-s001.zip › Supplementary Fgures.pdf]
